# Distinguish different sensorimotor performance of the hand between the individuals with diabetes mellitus and chronic kidney disease through deep learning models

**DOI:** 10.3389/fbioe.2024.1351485

**Published:** 2024-02-29

**Authors:** Pu-Chun Mo, Hsiu-Yun Hsu, Cheng-Feng Lin, Yu-Shiuan Cheng, I-Te Tu, Li-Chieh Kuo, Fong-Chin Su

**Affiliations:** ^1^ Department of Biomedical Engineering, College of Engineering, National Cheng Kung University, Tainan, Taiwan; ^2^ Department of Physical Medicine and Rehabilitation, National Cheng Kung University Hospital, College of Medicine, National Cheng Kung University, Tainan, Taiwan; ^3^ Department of Occupational Therapy, College of Medicine, National Cheng Kung University, Tainan, Taiwan; ^4^ Department of Physical Therapy, College of Medicine, National Cheng Kung University, Tainan, Taiwan; ^5^ Division of Nephrology, Department of Internal Medicine, Chi Mei Medical Center, Tainan, Taiwan; ^6^ Medical Device Innovation Center, National Cheng Kung University, Tainan, Taiwan

**Keywords:** sensorimotor function, deep learning, diabetes mellitus, chronic kidney disease, human biomechanics, bioengineering

## Abstract

Diabetes mellitus and chronic kidney disease represent escalating global epidemics with comorbidities akin to neuropathies, resulting in various neuromuscular symptoms that impede daily performance. Interestingly, previous studies indicated differing sensorimotor functions within these conditions. If assessing sensorimotor features can effectively distinguish between diabetes mellitus and chronic kidney disease, it could serve as a valuable and non-invasive indicator for early detection, swift screening, and ongoing monitoring, aiding in the differentiation between these diseases. This study classified diverse diagnoses based on motor performance using a novel pinch-holding-up-activity test and machine learning models based on deep learning. Dataset from 271 participants, encompassing 3263 hand samples across three cohorts (healthy adults, diabetes mellitus, and chronic kidney disease), formed the basis of analysis. Leveraging convolutional neural networks, three deep learning models were employed to classify healthy adults, diabetes mellitus, and chronic kidney disease based on pinch-holding-up-activity data. Notably, the testing set displayed accuracies of 95.3% and 89.8% for the intra- and inter-participant comparisons, respectively. The weighted F1 scores for these conditions reached 0.897 and 0.953, respectively. The study findings underscore the adeptness of the dilation convolutional neural networks model in distinguishing sensorimotor performance among individuals with diabetes mellitus, chronic kidney disease, and healthy adults. These outcomes suggest discernible differences in sensorimotor performance across the diabetes mellitus, chronic kidney disease, and healthy cohorts, pointing towards the potential of rapid screening based on these parameters as an innovative clinical approach.

## 1 Introduction

Globally, diabetes mellitus (DM) has significantly impacted healthcare costs and socioeconomic burdens, escalating from 966 billion United States dollars (USD) in 2021 to a projected 1,054 billion USD by 2045. A recent epidemic report estimated the global prevalence of diabetes at approximately 10.5%, set to rise to 12.2% by 2045 (Sun et al., 2022). Similarly, a recent epidemiological report indicated that the prevalence of chronic kidney disease (CKD) was 10.0% in adult populations globally; however, this value may be underestimated. Akin to DM-related impacts, CKD-related healthcare stands as the primary driver of medical and social costs in most countries (Sundström et al., 2022). Patients with severe DM often progress to CKD, which can impose greater care challenges; hence, CKD could be considered a more severe condition in patients with DM (Parving et al., 2006; Thomas et al., 2016). To monitor DM progression and prevent the development of severe disease, a low-cost, quick, and noninvasive method is needed.

Individuals with DM and CKD commonly experience peripheral nerve disorders (Baumgaertel et al., 2014), particularly peripheral neuropathy (Pop-Busui et al., 2017; Ezzeldin et al., 2019; Feldman et al., 2019; Karlsson et al., 2019). Clinical symptoms associated with neuropathies include pain, impaired thermal discrimination, sensory deficits, reduced motor function, and diminished or absent distal reflexes (Callaghan et al., 2015; Pop-Busui et al., 2017; Ezzeldin et al., 2019; Feldman et al., 2019). These symptoms significantly impact daily activities and could be critical in pre-DM and early stages of CKD (Singleton et al., 2001; Ziegler et al., 2008; Im et al., 2012; Smith and Singleton, 2012; Bongaerts et al., 2013; Asghar et al., 2014; Moorthi et al., 2019). Monitoring changes in sensorimotor function, an evident neurological feature, might be a feasible strategy to monitor the progression of both diseases. Early detection of these diseases could aid patients and clinicians in comprehending disease progression and subsequently achieving improved prognosis (Tesfaye et al., 2010; Bernardi et al., 2011; Bril et al., 2011; Dyck et al., 2011; Kempler et al., 2011; Spallone et al., 2011; Finnerup et al., 2015; Pop-Busui et al., 2017). Despite easily identifying neuropathies in both groups, the mechanism of neuropathies in DM and CKD remains unclear and may result in different neuropathic symptoms or sensorimotor features in these two diseases (Biessels et al., 2014; O Brien et al., 2014; Vincent et al., 2011; Zenker et al., 2013). For example, patients with CKD were found to exhibit poorer light touch sensory function than non-CKD participants, even after excluding the effects of DM (Moorthi et al., 2019). In other words, sensorimotor performance might vary between DM and CKD, despite both having neuropathies as diagnoses. Due to potential differences in sensorimotor performance between DM and CKD, evaluating sensorimotor function to monitor disease progression could prove to be a valuable, low-cost method.

Recent studies introduced a novel pinch-holding-up-activity (PHUA) test, using sensorimotor function measurements with robust psychometric properties (Chiu et al., 2009). These investigations aimed to discern disparities in hand sensorimotor performance hand between patients with DM and healthy adults (Chiu et al., 2014; Hsu et al., 2015), as well as differences between patients with CKD and healthy adults (Tu et al., 2019). These studies showed significant differences in sensorimotor performance between healthy adults and patients with peripheral neuropathic hands. Sensorimotor parameters—such as force ratio and percentage of maximal pinch force—were notably larger in patients with neuropathy, indicating that the use of inefficient or improper hand performance strategies. Furthermore, these parameters displayed medium-to-high correlations between sensory conditions and fine motor function (Shieh et al., 2011; Hsu et al., 2013).

However, these studies primarily relied on a limited set of parameters—such as force ratio or maximal pinch ratio—derived from specific events within the signals to determine the inferior sensorimotor performance in individuals with neuropathic hands. Unfortunately, these parameters proved insufficient for distinguishing sensorimotor features between the DM and CKD groups using current analytical approaches.

In recent years, machine learning has rapidly developed for human motion analysis. For fundamental research, Liu et al. proposed several base studies for extracting interpretable and explainable features to help build machine learning models for human activity recognition (HAR) (Liu et al., 2021; Hartmann et al., 2022; Liu et al., 2023) and published a feature extraction library for time-series data (Barandas et al., 2020). Hartmann et al. also found that high-level and interpretable features can be used in few-shot learning, and the results were promising (Hartmann et al., 2023). At the same time, a new branch in machine learning, called deep learning, has found widespread use in complex and noisy signal applications, where conventional analyses might struggle to extract pertinent information (LeCun et al., 2015; Goodfellow et al., 2016). Ideal deep learning models use original data or parameters without preprocessing or human-selected procedures. Previous studies have demonstrated the efficacy of convolutional neural networks (CNN) in appropriately handling time-series images or signals of human motion (Hannink et al., 2016; Hannink et al., 2017; Kautz et al., 2017). Architectures like VGG (Hannink et al., 2016; Hannink et al., 2017; Kautz et al., 2017), ResNet (Wang et al., 2017; Cheng et al., 2021), and dilation CNN (Arık et al., 2017; Bai et al., 2018; Lei et al., 2019) have emerged as strong candidates for processing time-series signals. Despite their demonstrated capabilities in processing time-series data, current studies rarely classify different peripheral neuropathies based on sensorimotor features of hand performance. Therefore, this study aimed to develop three distinct CNN models for classifying DM and CKD diagnoses based on hand sensorimotor function.

## 2 Materials and methods

### 2.1 Study participants

**FIGURE 1:**
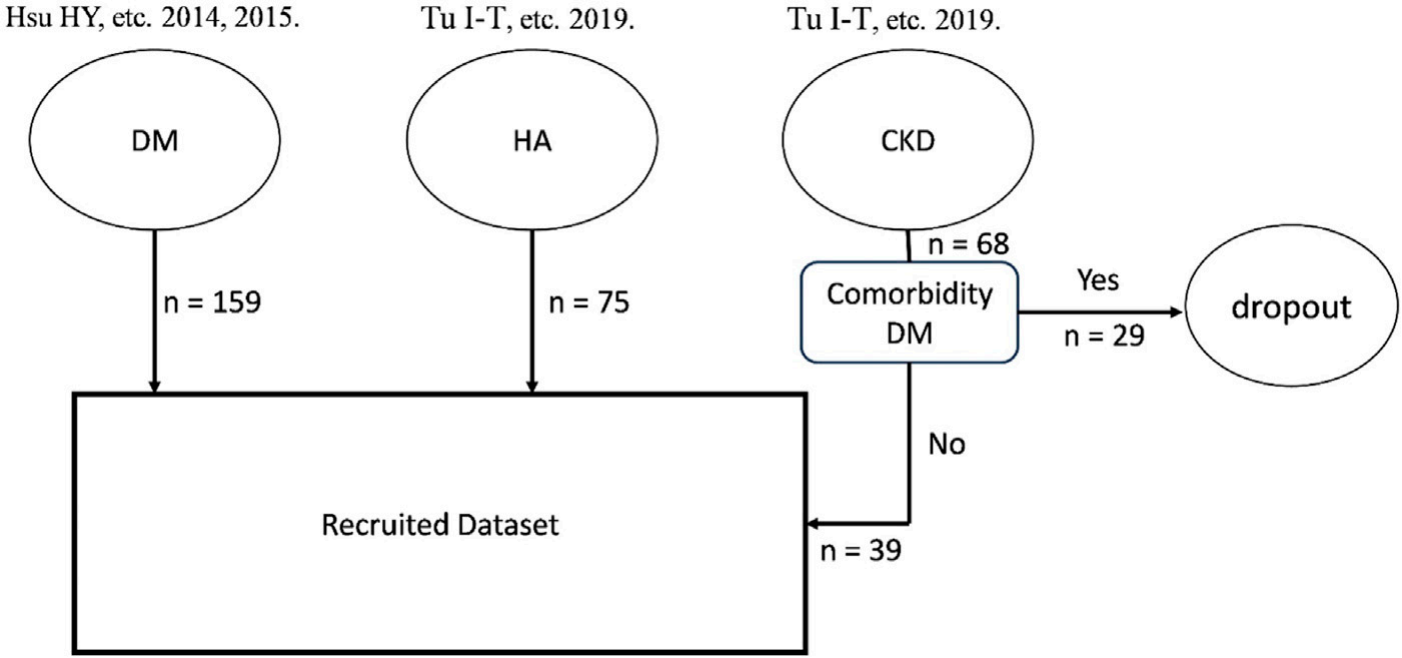
The flowchart for the dataset inclusion and exclusion from previous research.

All the data used in this study were retrospective and anonymized from previous research (Chiu et al., 2014; Hsu et al., 2015; Tu et al., 2019). Participant demographics from these studies are shown in Table 1. Sensorimotor function data were collected from participant hands between 2006 and 2018 using a standardized device and measurement protocol. The flowchart for the sampling inclusion and exclusion is described in Figure 1. The datasets included hand data from healthy controls and DM groups for both hands, whereas the dataset of the CKD group contained data solely from hand without a venous fistula. Neuropathies in DM and CKD arise from metabolism issues, including poor blood glucose control and the presence of toxic substance in blood. As a result, damage to neurons on both the right and left side is expected to be equal. Consequently, data from the right and left hands were assumed to be similar and were not segregated during training and testing. Inclusion criteria for DM followed the diagnostic guidelines of the American Diabetes Association in 1997, whereas all CKD participants were stage-5 (GLR <15 mL/min) and undergoing hemodialysis. To prevent complexities arising from comorbid conditions, participants with both DM and CKD were excluded. The control group exclusion criteria were as follows: (Sun et al., 2022): upper limb nerve injuries; (Sundström et al., 2022) acquired or congenital hand or wrist anomalies; (Parving et al., 2006) skin infections or diseases; (Thomas et al., 2016) diagnoses of DM, CKD, or any cardiovascular disease; (Baumgaertel et al., 2014) grade ≥2 arterial hypertension; and (Ezzeldin et al., 2019) cognitive dysfunction, and an inability to follow instructions. Informed consent was obtained from all participants, and the study adhered to the instructions of the Institutional Review Boards of Chi Mei Medical Center and Chiayi Christian Hospital (Chiu et al., 2014; Hsu et al., 2015; Tu et al., 2019).

**TABLE 1:** The demographic information of the three groups of participants.

		Control (*n* = 75)	DM (*n* = 159)	CKD (*n* = 37)
Age (years)		46.47 (16.28)	58.83 (9.61)	60.19 (9.31)
Onset (years)		—	9.55 (6.53)	5.44 (3.79)
Dominant (R/L)		72/3	159/0	37/0
Gender (M/F)		30/45	83/76	22/15
Total Samples in dataset		1,427	1,421	415
Intra	Training	827 (58%)	771 (54%)	251 (60%)
	Validation	300 (21%)	325 (23%)	82 (20%)
	Testing	300 (21%)	325 (23%)	82 (20%)
Inter (ratio%, cases)	Training	1,033 (72%, 55)	1,048 (74%, 116)	320 (77%, 28)
	Validation	166 (12%, 9)	168 (12%, 20)	40 (10%, 4)
	Testing	228 (16%, 11)	205 (14%, 23)	55 (13%, 5)

### 2.2 Instruments and data-collecting protocols

Sensorimotor hand function data were collected using a custom designed apparatus comprising a specific device (size: 6.0*4.5*9 cm, weight: 480 g), incorporating a six-axes loadcell (Nano-25, ATI Industrial Automation, Apex, NC) and tri-axial accelerometer (Model 2,412, Silicon Designs, Inc., Issaquah, WA) for the PHUA test. Previous studies have affirmed the validity and reliability of the PHUA test in assessing hand sensorimotor performance (Chiu et al., 2009; Shieh et al., 2011; Hsu et al., 2013; Chiu et al., 2014; Hsu et al., 2015; Tu et al., 2019). The load cell and accelerometer were set to a sampling rate of 100 Hz. The data collection protocols for PHUA were standardized across the three groups. Participants were instructed to: (Sun et al., 2022): pinch the device with the thumb and index fingertips, (Sundström et al., 2022), lift the device approximately 5 cm above the table and maintain for 5 s, (Parving et al., 2006), lift the device to approximately 30 cm at a self-determined speed, and (Thomas et al., 2016) slowly lower the device after 10 s. Each PHUA trial lasted approximately 15 s, with only the initial 10 s used for data collection to minimize bias during uncontrolled lowering periods. Each participant performed ten trials for each hand. The demographic information of the three groups of participants, final sample size, and the dataset after splitting are summarized in Table 1.

### 2.3 Dataset and preprocessing

In the collection of medical signals, resampling one participant is a common strategy for expanding the sample size. To resample, each of our subjects were asked to repeat the same protocol to generate the data samples. In this study, the PHUA data of one participant was resampled four to ten times. For the DM, data collection was resampled four to six times. For the healthy adults and CKD, the resampling was nine to ten times more than DM due to fewer participants and an imbalanced dataset. Although this method easily increases the sample size, it often leads to overfitting and data leakage. To prevent these issues and ensure model robustness, two different dataset splitting methods were employed: (Sun et al., 2022) inter-participants: the dataset was split based on participants, ensuring that the model did not encounter repeated participants during training, validation, or testing; and (Sundström et al., 2022) intra-participants: data splitting occurred within the trial of each participant. Each participant data were segregated for training, validation, and testing, ensuring that trials did not overlap across these phases. No other methods were used to expand the dataset used in this study.

The dataset was arranged for testing first, and then the remaining data were utilized for training and validation. The percentage of each group differed between the two data-splitting methods. When employing the inter-subject method to split the dataset, variations in the number of resample trials per subject could have led to slight differences in sample numbers. The sample sizes for training, validation, and testing are provided in Table 1. All hyperparameters and model structures were adjusted during the cross-validation phase. Once optimal validation results were obtained, the entire training and validation dataset were combined for final model training. Model performance was then evaluated using the dedicated testing set, without any modifications to the model to prevent data leakage.

### 2.4 Algorithm development and evaluations

Due to the PHUA protocol, we assumed that the features are present during the rising phase, and the time point of the rise is not restricted to every subject. Consequently, the features of the obtained signals in this study were presumed to exhibit time-translation symmetry and can be regarded as local features. The assumption of time-translation symmetry implies that the features would have similar forms but can appear any time without strict constraints. Under this assumption, the CNN structure is a suitable choice for searching for features over time by shifting the windows on the signal. Recent research has demonstrated the superiority and efficiency of CNN architectures over recurrent neural networks for analyzing time-series signals (Pascanu et al., 2012; Pascanu et al., 2013; Längkvist et al., 2014; Fawaz et al., 2019). Therefore, CNN models were constructed for three groups: healthy adults, DM, and CKD. The first model is derived from the VGG model (Simonyan and Zisserman, 2014), which uses a linear structure without shortcuts. The benefits of the VGG-like model (fully convolutional network [FCN]) offers simplicity and ease of comprehension but has limited depth. Its straightforward structure and fewer parameters make it a widely used option for testing deep learning models. The second model is based on ResNet (He et al., 2016). ResNet is structured with a shortcut, allowing residuals to traverse through the shortcut and learn, despite the network being extremely deep. A deep model is sufficiently robust for approximating a wide array of functions (Lin and Jegelka, 2018). The third proposed model, a dilation CNN (dil-CNN), represents the latest advancement capable of handling exceptionally long time-series data (Bai et al., 2018; Lei et al., 2019). This architecture compels nodes to glean essential features and transmit them through lengthy time series without necessitating a deeply layered design. In this study, we introduced FCN, ResNet, and dil-CNN architectures to distinguish among healthy adults and patients with DM and CKD.

**FIGURE 2:**
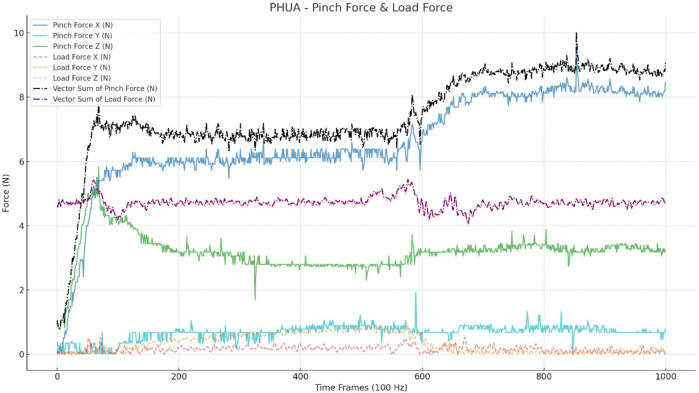
Input data for CNN models. This figure shows a trial of a healthy adult. The input includes a three-axis pinch force (Nt) and three-axis load force (Nt), which is calculated through the accelerations (a*0.48*9.8). The vector sum was calculated from the three axes of the pinch and load forces.

The input data are raw data with absolute values from 0 to 10 s (100 Hz), comprising 1,000 frames. To aid the model’s convergence, the pinch force vector sum and load force vector sum are included in the input. No other pre-processing or human-selected features were utilized for the model input. The trial sample is depicted in Figure 2.

**FIGURE 3:**
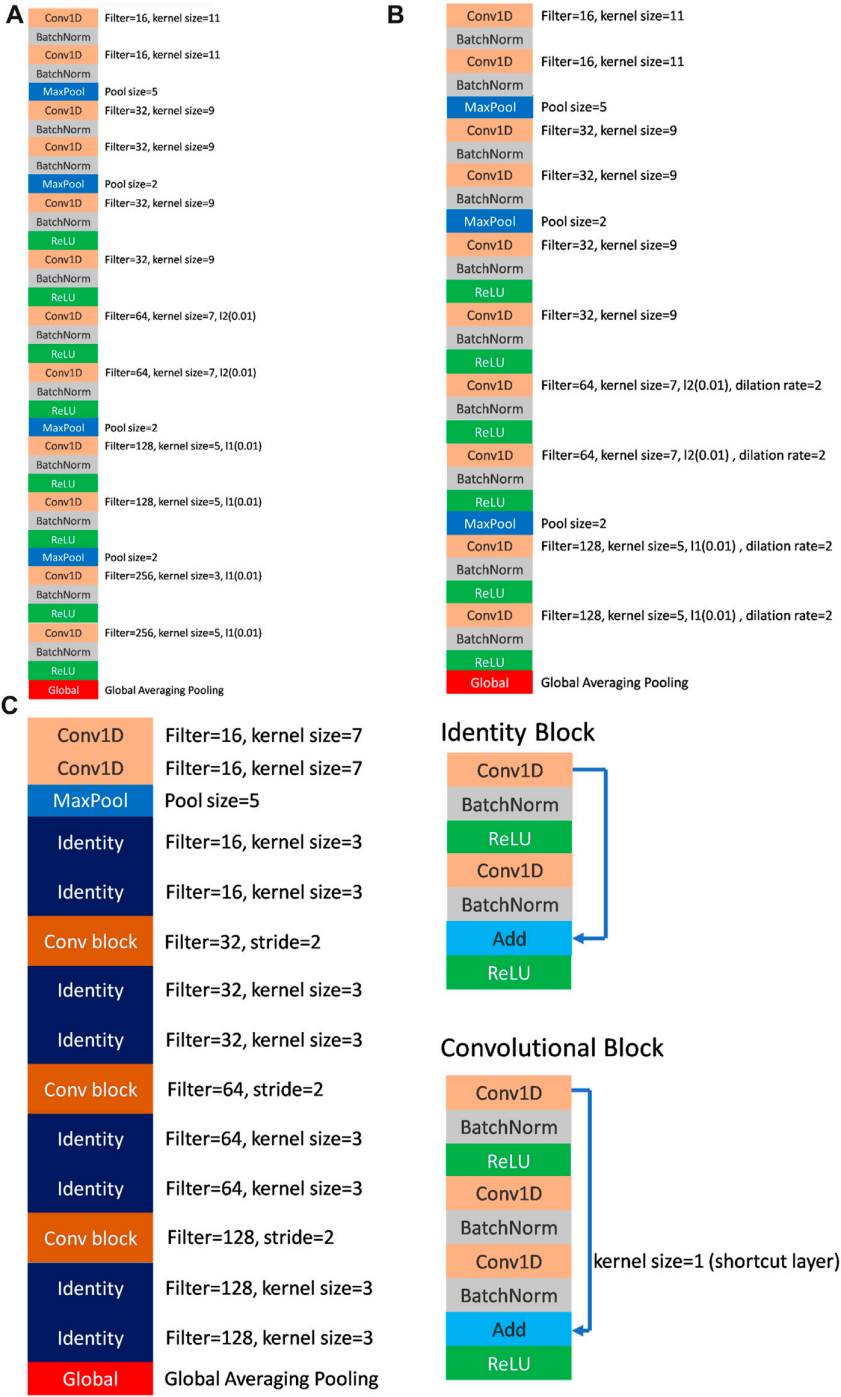
**(A)** The architecture of FCN (VGG-like) model. **(B)** The architecture of dil-CNN (dilation CNN) model. **(C)** The architecture of ResNet model and the details of identity block and convolutional block.

The model architecture is illustrated in Figure 3. The receiver operating characteristic curve (ROC) curve and area under the ROC curve (AUC) were used to evaluate model performance. The F_1_ score and confusion matrix were calculated to provide a comprehensive evaluation of accuracy and detailed category-specific performance.

The optimizer of CNN was RMSProp (lr = 0.0001, rho = 0.9, epsilon = 1e-7), and the L1 and L2 regularization was used for the parameters in the CNN models. A SoftMax function is used to output layer for deciding the category.

The CNN models were built by Python 3.9.9 and based on TensorFlow 2.6. The hardware for training and programming was i7-9,700, GTX3080 with 10 GB G and 40 GB RAM. The operating system was Windows 10.

The accuracy, confused matrix, ROC, and AUC are shown to evaluate the model performance. Also, the weighted F1 score is calculated in this study. Considered as the imbalance dataset, the F1 score with sample-weighted could show more information. All training, validation, and testing evaluation results were shown and discussed to display the overfitting situations.

## 3 Results

The model parameters used to demonstrate the model efficiency are listed in Table 2. In the FCN model, the total parameters employed were 502,979, whereas dil-CNN, shallower in structure, used 205,123 parameters. In ResNet, the total parameters is 379,907 (Table 2). The model structures of the FCN and dil-CNN were similar; however, the FCN was deeper, with two additional convolutional layers. The FCN and dil-CNN differed in its dilation rate, increasing from 2 to 4 in the seven–10 convolutional layers (Figures 3A, B). ResNet, following the structure of the original study with two blocks (He et al., 2016) repeated four times (Figure 3C). To mitigate overfitting risks, our models were designed with reference to the original article were not replicated at the same depth, such as VGG-19 or ResNet-34, we opted for a shallower architecture during the validation phase, refining it iteratively through trial and error.

**TABLE 2:** The parameters of the CNN models.

	VGG	ResNet	Dil-CNN
Trainable Parameters	500,867	376,643	204,035
Non-trainable Parameters	2,112	3,264	1,088
Total Parameters	502,979	379,907	205,123

The final test results indicate intra-participant accuracies for FCN, ResNet, and dil-CNN as 0.926, 0.874, and 0.953, respectively. Meanwhile, inter-participant accuracies for FCN, ResNet, and dil-CNN stand at 0.879, 0.875, and 0.898, respectively (Table 3). The weighted F1 scores (wF1) for intra-participant assessments of these models were 0.927, 0.873, and 0.953, whereas the inter-participant wF1 scores were 0.877, 0.869, and 0.897, respectively (Table 3).

**TABLE 3:** The evaluation results of the CNN models.

		Intra-subjects		Inter-subjects	
		Accuracy	wF1 score	Accuracy	wF1 score
Train	VGG	0.991	0.991	0.999	0.999
	ResNet	0.984	0.984	0.974	0.974
	Dil-CNN	0.999	0.999	0.999	0.999
Valid^a^	VGG	0.902 (0.018)	0.902 (0.019)	0.868 (0.050)	0.866 (0.052)
	ResNet	0.844 (0.022)	0.843 (0.023)	0.832 (0.058)	0.829 (0.065)
	Dil-CNN	0.926 (0.024)	0.925 (0.025)	0.901 (0.038)	0.901 (0.040)
Test	VGG	0.926	0.927	0.879	0.877
	ResNet	0.874	0.873	0.875	0.869
	Dil-CNN	0.953	0.953	0.898	0.897

**FIGURE 4:**
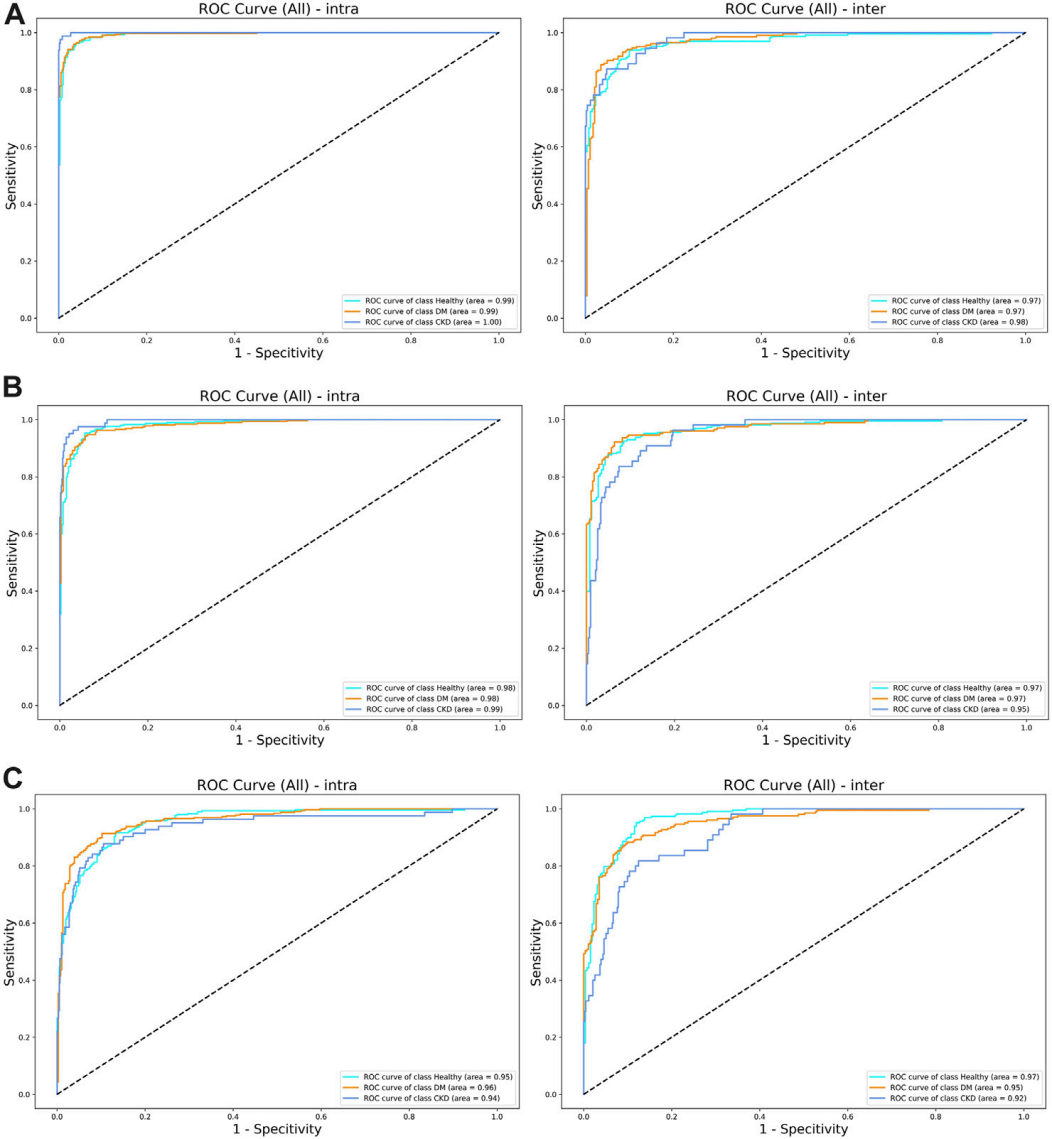
**(A)** The ROC curve of (left) intra-subjects and (right) inter-subjects of the del-CNN model. **(B)** The ROC curve of (left) intra-subjects and (right) inter-subjects of the VGG model. **(C)** The ROC curve of (left) intra-subjects and (right) inter-subjects of the ResNet model.

The evaluation of the model performance using the AUC is shown in Table 4; Figure 4. Across the intra-participant dataset, the AUCs for healthy adults were 0.984, 0.954, and 0.993 for FCN, ResNet, and dil-CNN, respectively. In the inter-participant dataset, these AUCs were 0.968, 0.966, and 0.966, respectively. The AUCs of the three models for DM in the intra-participant dataset were 0.983, 0.961, and 0.994, respectively. Assessing DM within the interparticipant dataset yielded AUCs of 0.972, 0.952, and 0.973. Finally, for CKD in the intra-participant dataset, AUCs were 0.995, 0.938, and 0.999. For the inter-participant dataset, the AUCs of the three models were 0.953, 0.916, and 0.977, respectively (Table 4).

**TABLE 4:** The Area Under the ROC Curve (AUC) of three groups under three CNN methods.

	VGG		ResNet		Dil-CNN	
	Inter	Intra	Inter	Intra	Inter	Intra
Healthy Adults (Control)	0.968	0.984	0.966	0.954	0.966	0.993
Diabetes Mellitus (DM)	0.972	0.983	0.952	0.961	0.973	0.994
Chronic Kidney Disease (CKD)	0.953	0.995	0.916	0.938	0.977	0.999

## 4 Discussion

The contemporary diagnosis of DM and CKD in clinical settings relies on blood tests (Harris and Eastman, 2000; Stevens and Levey, 2009), considered the golden standard. However, the proposed PHUA test and the findings in this study do not aim to replace this gold standard in clinical practice. Instead, the PHUA test, combined with a deep learning model, offers a quicker and less invasive method for distinguishing between patients with DM and CKD in medical scenarios that require rapid screening or in places where laboratory examinations are not readily available. Our findings suggest that PHUA with a deep learning model could potentially discern differences in the sensorimotor features of the hand between CKD and DM. Therefore, this study may suggest a simple test to aid in consistently monitoring the progression of DM and preventing its advancement to CKD (Parving et al., 2006; Thomas et al., 2016). Previous studies demonstrated that regular follow-up examinations can reduce the severity of complications, particularly for individuals >45 or <45 but with significant risk factors such as obesity and a family history of DM (Pippitt et al., 2016). Assessing sensorimotor capability is typically simple and rapid in clinical, community, or home-based scenarios. To mitigate evaluation difficulties and potential inter-tester errors, the PHUA could serve a suitable apparatus for clinical assessments due to its well-define design rationale. Its design was developed to challenge participant reflex motor responses while performing upward movements by pinching a glossy surface (Chiu et al., 2009; Hsu et al., 2009; Shieh et al., 2011). The findings of this study indicate that the PHUA test, coupled with a deep learning model, could serve as a potential tool to evaluate the sensorimotor function of the hands, and differentiate between DM and CKD based on sensorimotor impairments, owing its high accuracy and AUC. Given its higher accuracy, our proposed model with the PHUA test could be a viable option for swift clinical screening and monitoring, particularly for subjects who may be neglecting risks while being away from medical providers.

**FIGURE 5:**
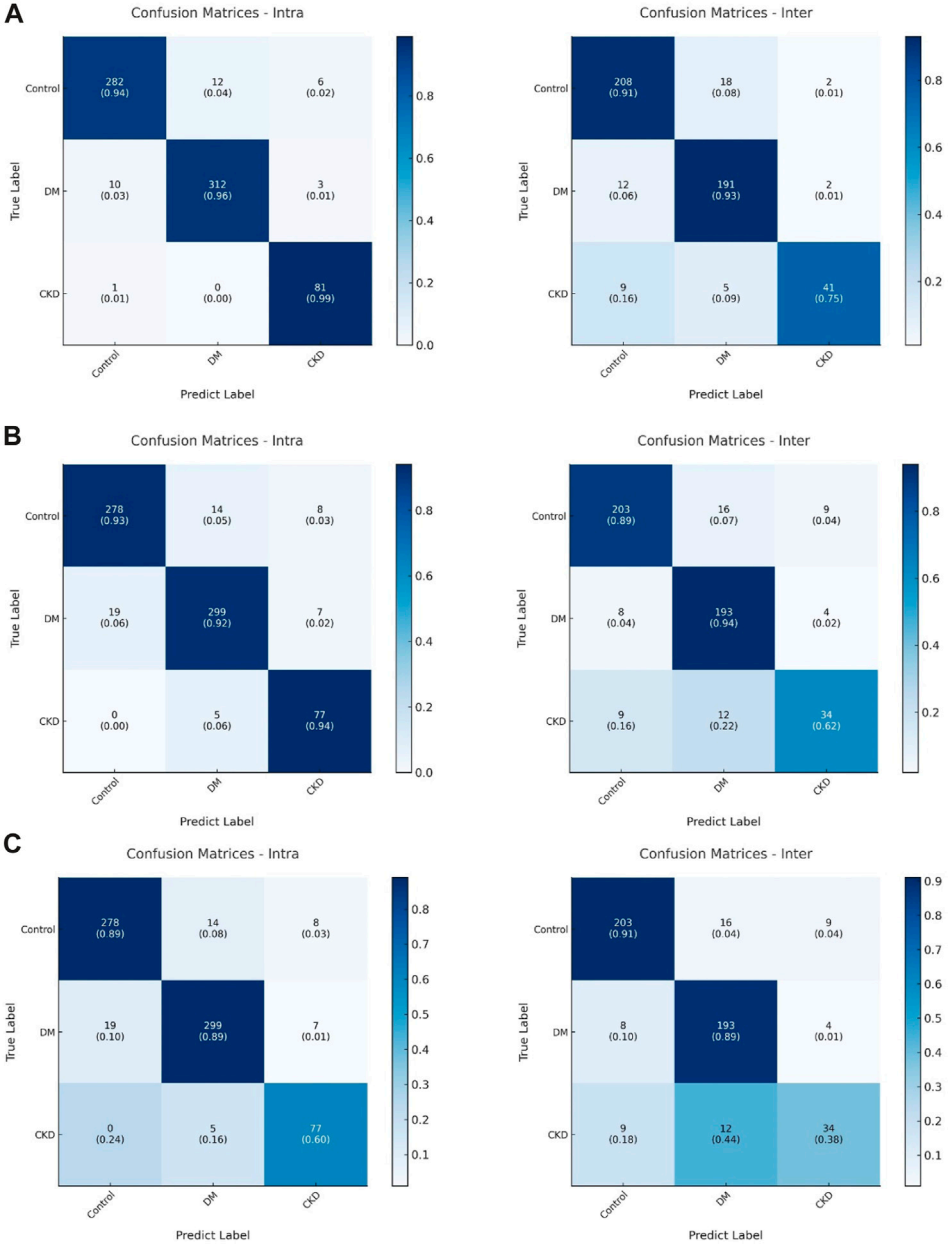
**(A)** The confusion matrix of (left) intra-subjects and (right) inter-subjects of the dil-CNN model. **(B)** The confusion matrix of (left) intra-subjects and (right) inter-subjects of the VGG model. **(C)** The confusion matrix of (left) intra-subjects and (right) inter-subjects of the ResNet model.

Neuropathy in both DM and CKD typically manifests as neural system damage that impairs sensorimotor performance (Tesfaye et al., 2010; Baumgaertel et al., 2014), but research on distinctions between DM and CKD in this context is limited. Our proposed models suggest that deep learning models can uncover variations in sensorimotor patterns between DM and CKD (Figure 5) and marked the sample numbers in Figure 5 because of the imbalanced sample size between different groups (Jannat et al., 2023). The confusion matrix generated by the dil-CNN model implies potential differences in PHUA-based sensorimotor performance between DM and CKD, aligning with previous findings (Moorthi et al., 2019). Earlier research demonstrated that patients with CKD exhibit poorer light touch sensation compared with healthy adults and those with DM (Moorthi et al., 2019). However, the impact of worsened sensory sensation on clinical evaluation of sensorimotor patterns remains unexplored. The study findings indicate that a robust model could discern specific differences in sensorimotor features between DM and CKD. Despite similarities in impaired sensorimotor function—such as sensory impairment and motor function deficit—between DM and CKD, this discovery suggests that the decline in sensorimotor function in these conditions might arise from different mechanisms or conditions. However, although the deep learning model highlights differences in sensorimotor patterns between DM and CKD, it remains a black box, unable to specify the exact degenerative processes causing these differences (Castelvecchi, 2016). Notably, sensory or motor function impairment affects PHUA performance (Chiu et al., 2009; Shieh et al., 2011; Hsu et al., 2012; Hsu et al., 2013). Previous studies have suggested that neuropathies and the underlying mechanisms causing sensorimotor degeneration in individuals with DM or CKD may differ (Tesfaye et al., 2010; Callaghan et al., 2015; Pop-Busui et al., 2017; Ezzeldin et al., 2019; Feldman et al., 2019; Karlsson et al., 2019). Neuropathy in DM stems from uncontrolled blood glucose levels, deforming blood vessels and leading to insufficient neuronal nourishment (Tabatabaei-Malazy et al., 2011; Baumgaertel et al., 2014). Damage primarily occurs in the distal body parts (Ezzeldin et al., 2019). In contrast, neuropathy in CKD results from toxic substances in the blood (Baumgaertel et al., 2014; Arnold et al., 2016), affecting nervous tissues throughout the body, including the muscles, neural system, and metabolic system (Arnold et al., 2016). Although these models solely differentiate motor patterns between DM and CKD, prior research suggests that differing neuropathic mechanisms also imply varying severity levels and affected the body system ranges (Moorthi et al., 2019).

During the model design and adjustment phase, a smaller kernel size resulted in higher accuracy within the modified model structure. This finding aligns with previous research suggesting that smaller kernels may lead to better convergence, a principle noted in the original VGG designer work on image recognition (Simonyan and Zisserman, 2014). The three proposed model architectures suffer from overfitting. During the design phase, regularization to prevent overfitting, such as the batch norm layer, L1 or L2 regularization (Krogh and Hertz, 1992; Schmidhuber, 2015), and dropout (Srivastava et al., 2014), was tested, and the overfitting condition did not improve. Moreover, ResNet displayed severe overfitting compared with the other dil-CNN models, but with more parameters included in the model (Table 2). This could be attributed to the shortcut structure of ResNet, as demonstrated in previous research where a neural network with shortcuts can fit signals effectively given enough layers (Lin and Jegelka, 2018). Although ResNet structure in this study was not extremely deep, overfitting persisted. Attempts were made to explore models with fewer layers, but they resulted in underfitting compared with the final proposed structure. Further research on shortcut-designed models for time-series sensorimotor performance data is warranted to address these challenges.

To accommodate the increased computational power and memory requirements of larger models, the final model included only six convolutional layers in the FCN and dil-CNN, whereas six identity blocks were used in ResNet. Notably, the last layer of average pooling outperformed the flattened layer (Lin et al., 2013). No additional dense layer followed global average pooling, a strategy that mitigated overfitting and increased testing accuracy. This aligns with the findings in deep learning model design for image recognition post-2013 (Lin et al., 2013; He et al., 2016; Szegedy et al., 2016).

The dil-CNN showed superior robustness with fewest parameters among compared models. The total number of parameters in the dil-CNN was 205,123, nearly half that of ResNet (Table 2) and one-third that of the FCN. The dil-CNN had a higher accuracy than the FCN (0.953 vs. 0.926 in intra-participant; 0.898 vs. 0.879 in inter-participant; Table 3). In the proposed model, dil-CNN exhibited the best performance. Leveraging the design of dilation in convolution, the dil-CNN effectively combines information across extended time-series data. In our study, the structure based on VGG remains unchanged, with dilation being the sole modification, yet it notably enhances accuracy. However, we did not evaluate the dilation kernel with a residual network (Bai et al., 2018) in our proposed models because of observed overfitting in ResNet. Future research should explore additional adjustments to the dil-CNN, potentially incorporating a shortcut connection. Nevertheless, for classification tasks involving sensorimotor signals, the dilation CNN might prove superior to other structures.

Previous studies have relied on several human-selected parameters, resulting in a lower AUC for patients with DM and healthy adults (AUC = 0.724) (Chiu et al., 2014), as well as for CKD and healthy adults (AUC = 0.848) (Tu et al., 2019), whereas the dil-CNN exhibited higher AUC values (AUC > 0.96, Table 4). Notably, complete raw data were included in our analysis, contributing to improved prediction accuracy and potential for handling multiple classes using deep-learning models, as indicated by our results.

Our study has several limitations that warrant acknowledgement. All proposed models in this study faced challenges related to overfitting, despite incorporating regularization and batch-normalization layers. This issue might stem from the restricted total number of participants, suggesting the need for a larger sample size, particularly for machine-learning models reliant on deep learning. Increasing the sample size could enhance model stability and mitigate overfitting concerns in real-world applications. In addition, few-shot learning with pre-processing and feature engineering (Hartmann et al., 2023) could be a possible solution for PHUA testing, especially using the model in following subjects with a high potential risk of DM and CKD. Moreover, the models based on deep learning represent black boxes in this study, lacking explicit explanations for distinguishing sensorimotor performance in DM from that in CKD. Although visualization methods such as Grad-CAM (Selvaraju et al., 2017), Grad-CAM++ (Chattopadhay et al., 2018), and I-GOS (Qi et al., 2019) exist to potentially elucidate model attention, these approaches offer visual insights without statistical significance and are susceptible to misleading interpretations (Subramanya et al., 2019). Developing interpretable AI warrants further investigation, involving visualization techniques like Grad-CAM, sensitivity analysis, and generative models. In the other way, human-selected features with interpretable feature engineering can be an innovative method for developing a explainable machine learning model (Hartmann et al., 2022; Hartmann et al., 2023). These efforts aim to assist clinical staff identifying meaningful features or key points for evaluating patient sensorimotor performance. Another limitation lies in the insufficient variety of diagnoses considered, limiting the model adaptability in clinical scenarios. Future studies should encompass diagnoses with similar sensorimotor impairments, such as carpal tunnel syndrome, peripheral neural damage, or neuromuscular disorders, to refine the model output accuracy. An additional limitation is that our study represents just the initial step toward rapid screening using PHUA with a DNN. Furthermore, participants with DM and CKD were exclusively recruited at severe stages. To establish a comprehensive clinical solution for rapid screening or sensorimotor evaluation, different stages and progressions of DM and CKD should be considered, ensuring model robustness across diverse patient profiles.

Our findings underscored the potential of PHUA coupled with a deep learning model to differentiate various sensorimotor patterns among healthy adults and patients with DM and CKD. For the evaluation of human sensorimotor performance based on time-series signals, the dilated CNN structure demonstrated notable accuracy and efficiency. Future studies require larger sample sizes encompassing varying disease severities and considerations for the comorbidity of DM and CKD to advance the next-generation model. Developing an interpretable model is crucial to facilitate its practical application in clinical settings.

In conclusion, our study highlights the capacity of the DNN model to distinguish between healthy adults, participants with DM, and CKD participants through the innovative motor performance evaluation tool, PHUA. PHUA integrated with the dil-CNN model exhibits remarkable stability and accuracy, presenting sensorimotor performance assessment as a novel approach to aid in evaluating CKD and DM diagnosis stages and offering an effective screening method for both disorders. This study presents an innovative application of machine learning in clinical evaluation, particularly for patients with DM and CKD.

## Data Availability

The original contributions presented in the study are included in the article/Supplementary material, further inquiries can be directed to the corresponding authors.

## Abbreviations

DM, diabetes mellitus; CKD, CKD chronic kidney disease; PHUA, pinch-holding-up-activity; HAR, human activity recognition; CNN, convolutional neural networks; FCN, fully convolutional network; ROC, receiver operating characteristic curve; AUC, area under the ROC curve; wF1, weighted F1 scores; VGG, VGG is a model name, naming after the Visual Geometry Group.
